# The Interferon-Induced Gene *Ifi27l2a* is Active in Lung Macrophages and Lymphocytes After Influenza A Infection but Deletion of *Ifi27l2a* in Mice Does Not Increase Susceptibility to Infection

**DOI:** 10.1371/journal.pone.0106392

**Published:** 2014-09-03

**Authors:** Mohamed A. Tantawy, Bastian Hatesuer, Esther Wilk, Leonie Dengler, Nadine Kasnitz, Siegfried Weiß, Klaus Schughart

**Affiliations:** 1 Department of Infection Genetics, Helmholtz Centre for Infection Research and University of Veterinary Medicine Hannover, Germany; 2 Department of Molecular Immunology, Helmholtz Centre for Infection Research, Hannover, Germany; 3 University of Tennessee Health Science Center, Memphis, Tennessee, United States of America; University of Pittsburgh, United States of America

## Abstract

Interferons represent one of the first and essential host defense mechanisms after infection, and the activation of the IFN-pathway results in the transcriptional activation of hundreds of interferon-stimulated genes. The alpha-inducible protein 27 like 2A (*Ifi27l2a*) gene (human synonym: *ISG12*) is strongly up-regulated in the lung after influenza A infection in mice and has been shown in gene expression studies to be highly correlated to other activated genes. Therefore, we investigated the role of *Ifi27l2a* for the host defense to influenza A infections in more detail. RT-PCR analyses in non-infected mice demonstrated that *Ifi27l2a* was expressed in several tissues, including the lung. Detailed analyses of reporter gene expression in lungs from *Ifi27l2a-LacZ* mice revealed that *Ifi27l2a* was expressed in macrophages and lymphocytes but not in alveolar cells or bronchiolar epithelium cells. The number of macrophages and lymphocyte strongly increased in the lung after infection, but no significant increase in expression levels of the LacZ reporter gene was found within individual immune cells. Also, no reporter gene expression was found in bronchiolar epithelial cells, alveolar cells or infiltrating neutrophils after infection. Thus, up-regulation of *Ifi27l2a* in infected lungs is mainly due to the infiltration of macrophages and lymphocytes. Most surprisingly, deletion of *Ifi27l2a* in mouse knock-out lines did not result in increased susceptibility to infections with H1N1 or H7N7 influenza A virus compared to wild type C57BL/6N mice, suggesting a less important role of the gene for the host response to influenza infections than for bacterial infections.

## Introduction

Every year, about 500 million people are infected by the influenza A virus worldwide, of which about 500,000 die [1]. In recent history, the emergence of new influenza subtypes has caused severe pandemics [2], [3], [4], the most severe one in 1918 caused about 30–50 million deaths worldwide [5]. Furthermore, a new variant of a seasonal H1N1 virus, pH1N1, caused a world-wide pandemic in 2009 [6], [7], [8]. In addition, the emergence of H5N1 and H7N9 underline the continuous thread of newly emerging subtypes that may cross species barriers and cause new pandemics in humans.

After infection with influenza A virus, the host activates a multitude of anti-viral responses to control virus replication, to activate the innate and adaptive immune response and eventually eliminate the pathogen. In the early phase, infected cells and immune cells detect specific pathogen-associated molecular patterns (PAMPs) via membrane-associated and intracellular pathogen recognition receptors (PRRs). Activation of PRRs results in stimulation of signaling pathways that lead to transcriptional activation of early response genes, such as interferons, chemokines and cytokines (reviewed in [9], [10], [11], [12], [13], [14], [15]). The interferons represent one of the most important host defense mechanisms against viral infections. *Ifnb1*, *Ifna* and Ifn-λ genes belong to the type I and type III interferons, respectively, that are produced mainly in infected cells and in plasmacytoid dendritic cells [16] early after infection. They subsequently bind to specific receptors and activate the interferon signaling pathway in infected as well as neighboring cells (reviewed in *e.g.* [11], [17], [18], [19], [20], [21], [22]). Stimulation of the IFN pathway results in the transcriptional activation of hundreds of interferon-activated genes [23], [24], also referred to as ISG (interferon-stimulated) or IFI (interferon induced) genes. ISGs and IFIs establish an anti-viral state that protects cells from infection and replication of pathogens (reviewed in *e.g.* [21], [23], [24], [25], [26], [27]). For some of the ISGs, the anti-viral function has been described, *e.g.* MX1, EIFAK2 (PKR, PRKR), IFITM3, ISG15, RSAD2 (Viperin), and OAS1 [28], [29], [30], [31], [32], [33], [34], [35], [36]. However, the functional role of the majority of ISGs remains to be elucidated.

In global lung transcriptome analysis of C57BL/6J mice and expression quantitative trait loci (eQTL) studies of pre-Collaborative Cross lines, the *Ifi27l2a* (alpha-inducible protein 27 like 2A) gene was found to be highly expressed after infection with influenza A virus [37], [38]. Gene expression studies and subsequent network analysis in lines of the pre-CC mouse population showed that *Ifi27l2a* was one of the genes exhibiting putative causative relationships with a large number of downstream connected genes [37]. The IFI27L2A protein is localized at the inner nuclear membrane in transfected cell lines [39], [40] where it sequesters and inhibits the function of the NR4A1 nuclear receptor. This in turn causes the down-regulation of NR4A1-target genes [40]. Other studies suggested that IFI27L2A represents a mitochondrial protein that sensitizes cells to apoptotic stimuli via mitochondrial membrane destabilization [41]. *Ifi27l2a* knock-out mice are more susceptible to bacterial infections [42].

Here, we investigated the expression and function of the IFN-induced *Ifi27l2a* gene during the course of influenza A virus infection. We describe the cell type-specific expression of *Ifi27l2a* in the lung and its role for the host defense to influenza A infections in *Ifi27l2a* knock-out mice. In the lungs of non-infected animals *Ifi27l2a* was strongly expressed in macrophages and also found in lymphocytes. After influenza infection a strong increase in overall expression levels of *Ifi27l2a* was observed in the lung. This increase was mainly due to infiltration of macrophages and lymphocytes. *Ifi27l2a* knock-out mice did not exhibit a more susceptible phenotype compared to wild type mice after infection with H1N1 or H7N7 influenza A virus.

## Results

### *Ifi27l2a* is expressed in several tissues and in lung leukocytes and increases after infection with influenza A virus

We first studied the expression of *Ifi27l2a* in non-infected mice. RT-PCR analyses detected transcripts in kidney, liver, lung, spleen and salivary gland whereas no expression was seen in heart (Figure S1 in File S1). We also re-analyzed previous microarray studies for global gene expression changes in the lung after influenza A infection [38]. *Ifi27l2a* was already expressed at a high level in lungs of non-infected mice and represents one of the most strongly up-regulated interferon-induced genes after infection. A strong increase in expression levels of *Ifi27l2a* was detected as early as two days post infection. Expression peaked from day 3 to day 8 post infection (p.i.) and then declined to baseline levels between day 14 and 18 p.i. (Figure S2 in File S1).

**Figure 1:**
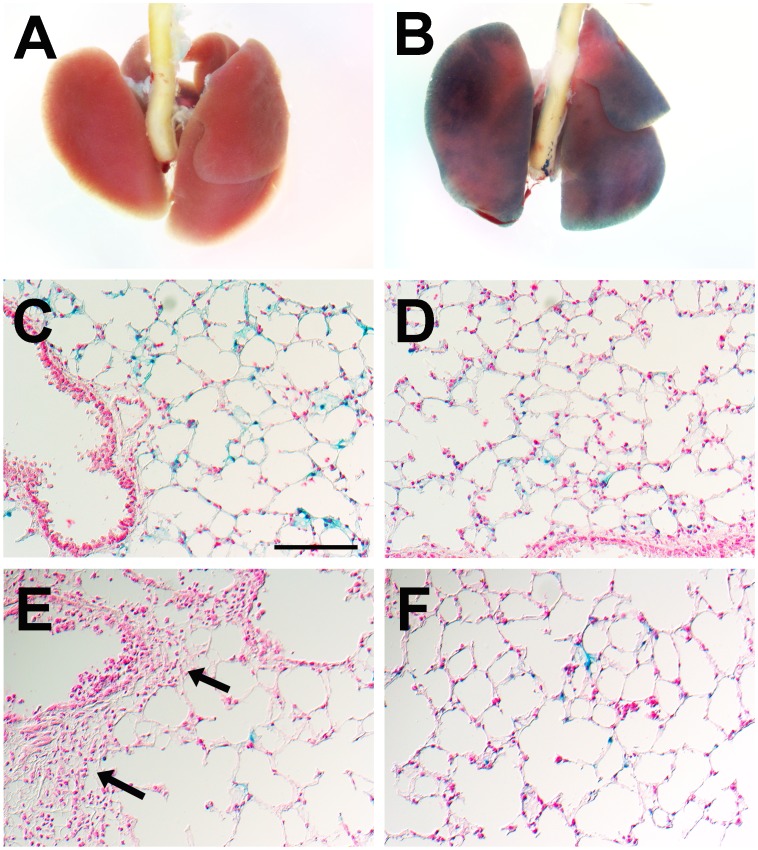
Analysis of LacZ reporter gene in *Ifi27l2a* knock-out mice reveals gene expression in lung macrophages in naïve and influenza-infected mice. The LacZ reporter gene allows following transcriptional regulation of the *Ifi27l2a* gene locus in the lungs mice. In whole mounts of *Ifi27l2a^-/-^* mice blue staining resulting from β-galactosidase activity was detected throughout the entire lung (B) whereas no staining was observed in lungs of C57BL/6N-*Ifi27l2a^+/+^* wild type mice (A). In lung cryosections, expression of the reporter gene was seen in macrophages in the alveolar regions of non-infected lungs (C,D). No staining of epithelial cells in the airways, alveolar regions and blood vessels was observed (C). After infection with 2×10^5^ FFU PR8 virus LacZ-positive cells were observed in alveolar regions (F) whereas no ß-galactosidase staining was found in infiltrates of peribronchiolar or perivascular regions (E, arrows) at day 3 p.i. At the cellular level, similar staining intensities before and after infections were observed (C-F). These results confirmed that *Ifi27l2a* is expressed in resident lung macrophages but also in the lung-infiltrating macrophages after influenza A virus infection. *Ifi27l2a* expression could not be detected in the immune cell infiltrates around broncheoli and blood vessels which contain mainly granulocytes. Scale bar (identical for all sections) in (C): 100 µm.

We further investigated *Ifi27l2a* gene expression at the cellular level using a *Ifi27l2a* knock-out mouse line that was generated by insertion of a LacZ reporter gene into the locus (Figure S3 in File S1). In this way, it was possible to follow transcriptional activation of the gene locus by studying expression of the reporter gene. After incubating lungs from *Ifi27l2a^-/-^* mice with the LacZ substrate X-Gal, blue staining was observed throughout the entire lung (Figure 1B) whereas no staining was observed in wild type animals (Figure 1A). In lung sections, expression of the LacZ reporter gene was observed in macrophages residing in alveolar regions of the lung (Figure 1C, D). No LacZ staining was seen in bronchi and bronchioli nor in blood vessels. After infection with influenza A virus PR8 (A/Puerto Rico/8/1934 [H1N1]) *Ifi27l2a^-/-^* mice exhibited more LacZ-positive macrophages at day 3 p.i. in alveolar regions (Figure 1E, F) whereas no staining was observed in peribronchial or in perivascular infiltrates (Figure 1E, F, arrows).

**Figure 2:**
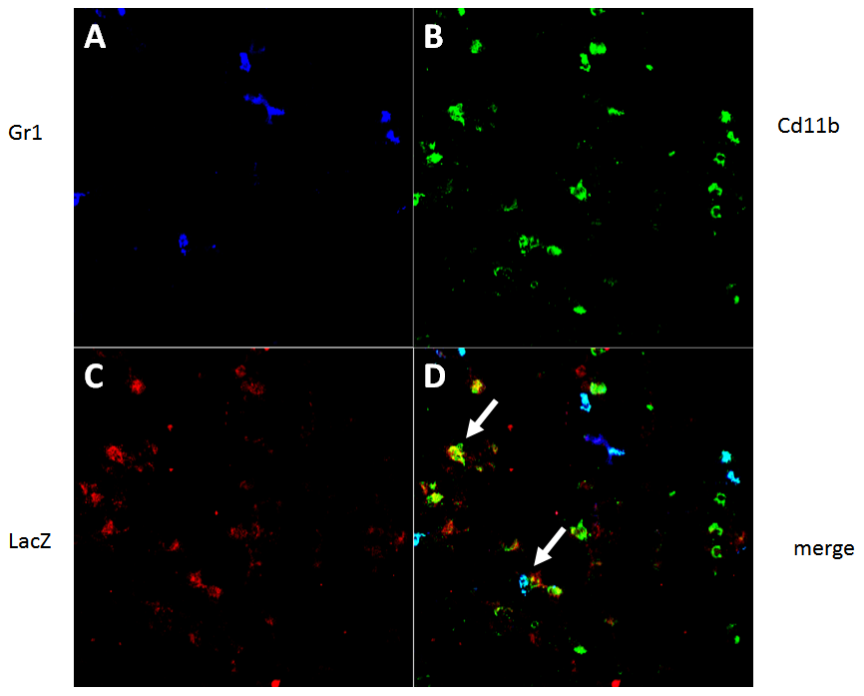
Immunofluorescent analysis of LacZ reporter gene expression and cell-specific marker genes. Lungs of PR8 infected (2×10^5^ FFU) *Ifi27l2a^-/-^* mice were prepared three days post infection and cryo-sections were co-stained with antibodies against Gr1 (A), CD11b (B) and LacZ (C). In the overlay picture, co-localization of LacZ and CD11b-stained macrophages was frequently observed (D). For CD11b/Gr1 double-positive neutrophils no co-immunostaining with LacZ could be detected.

To further confirm these observations, we performed immunofluorescent staining of infected lungs from *Ifi27l2a* knock-out mice for macrophages, granulocytes or alveolar epithelial cells. We observed a co-localization of the LacZ-reporter gene together with CD11b antibodies confirming *Ifi27l2a* expression in macrophages (Figure 2). However, no co-expression of LacZ reporter gene and CD11b/Gr1 double-positive cells (representing granulocytes) was observed (Figure 2). Also, no co-localization with epithelial cell adhesion molecule (Epcam) could be detected (data not shown).

**Figure 3:**
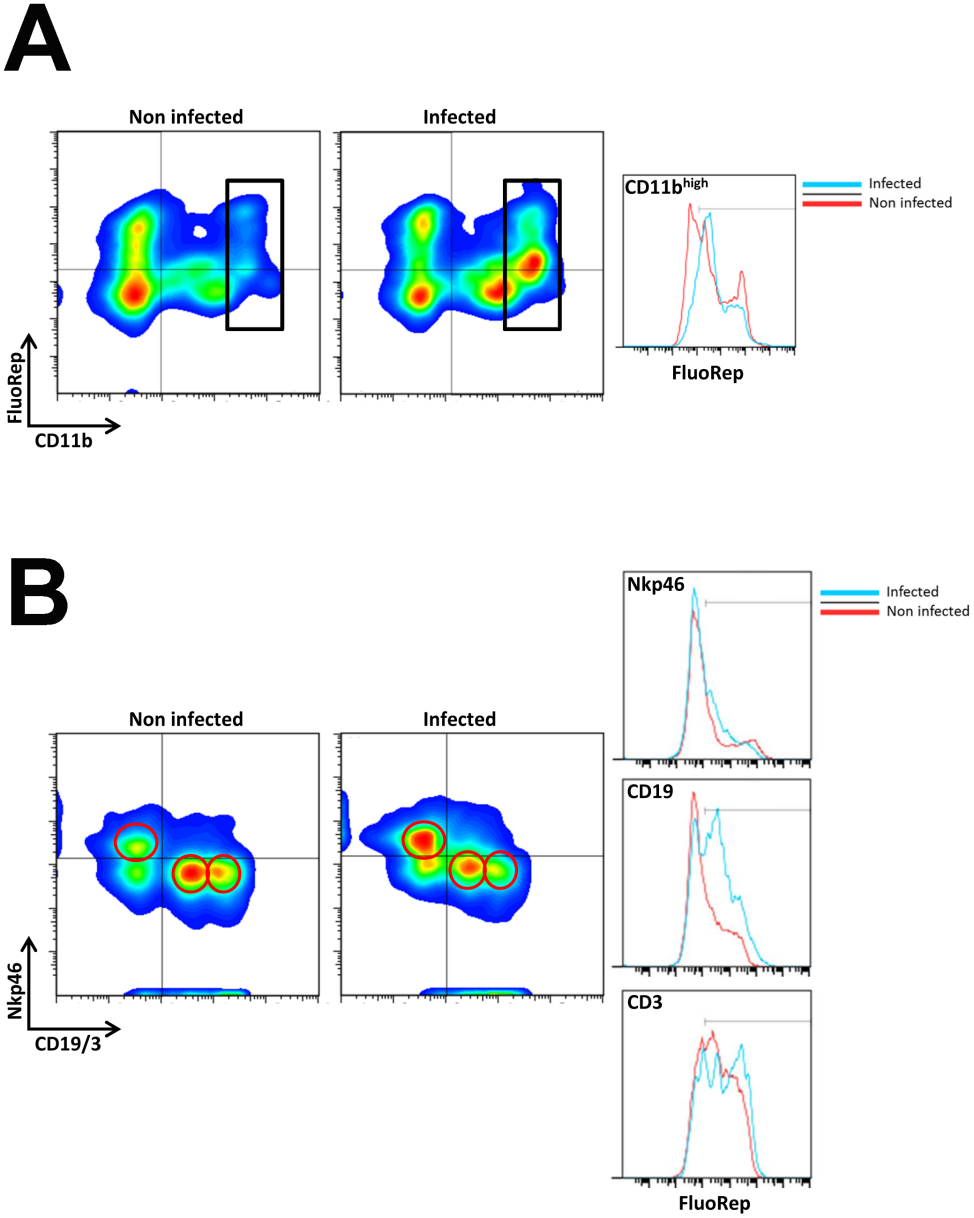
Flow cytometric analysis of immune cells in the lung revealed LacZ reporter gene expression in macrophages and lymphocytes. Cells were prepared from lungs of naïve and PR8 infected (2×10^5^ FFU) female mice (3dpi). Different immune cell populations were identified by gating on the respective positive population: CD11b^high^ cells (macrophages) were discriminated within the leucocyte population as depicted in (A). Lymphocytes were distinguished by using αNKp46 (NK cells), αCD3 (T cells) and αCD19 (B cells). Antibodies staining CD19 and CD3 were labeled with fluorochromes APC and Alexa 647, respectively. They were detected in the same channel forming distinct populations as verified by independent staining (data not shown). The left population in the lower right quadrant represents αCD19-APC with lower fluorescence intensity whereas the CD3 positive population is reflected in the right population with higher fluorescence intensity (B). All immune cell populations as indicated by gates in the density plots exhibited a distinct expression of the LacZ reporter gene. Red circles represent the respective gated cell populations. FluoRep: staining intensity using fluorescein di β-D-galactopyranoside (FDG) as substrate for ß-galactosidase (see Material and Methods for details). Red line in histograms indicates cells from naïve, blue line from infected mice. For group size, see Table 1.

**Figure 4:**
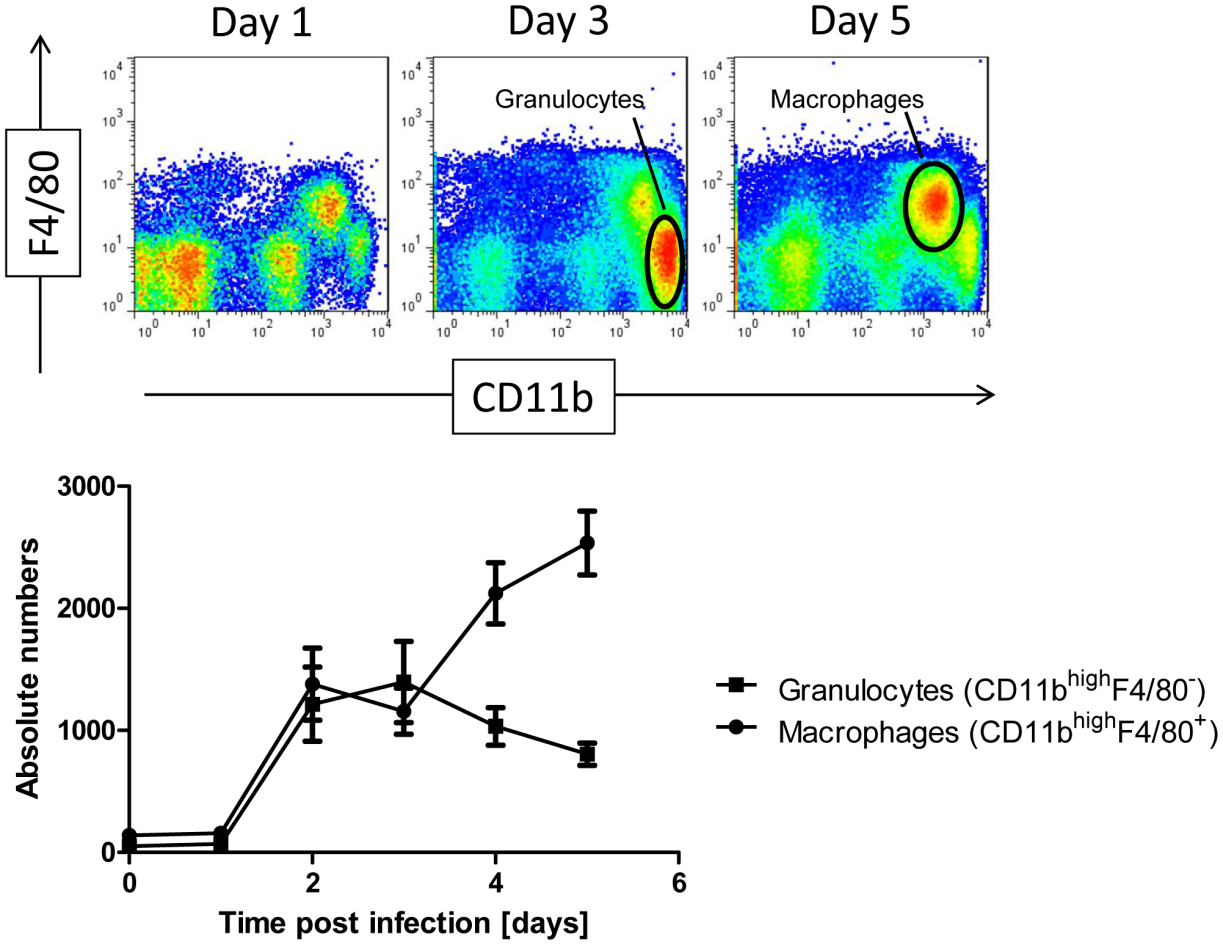
Flow cytometric analysis of immune cells in lungs showed an increase of macrophages and granulocytes after infection. Female C57BL/6J mice were infected with 2×10^3^ FFU PR8 (H1N1) virus and cellular infiltrates were determined by flow cytometry. The total number of granulocytes and macrophages strongly increased after infection. Absolute numbers were determined based on a standard curve of CD45^+^ cells after influenza infection equalizing the individual measurements to the same amount acquired.

Furthermore, we determined the immune cell populations in lungs before and after infection using fluorescein di β-D-galactopyranoside (FDG) as substrate by flow cytometry. Reporter gene activity was observed in lung macrophages (Figure 3 A), T, B and NK cells (Figure 3B) in non-infected lungs. Within the leukocyte population, 59% of macrophages, 26% of B cells, 65% of T cells, and 28% of NK cells exhibited positive LacZ-staining, respectively (Table 1). After infection with influenza A virus, the total number of granulocytes and macrophages increased strongly after infection (Figure 4). The macrophage population increased on day 3 compared to non-infected controls from 13% to 32% (Table 1, Figure 3). The lymphocytic populations relatively decreased at this time. The percentage of LacZ-positive cells increased in the macrophage population from 59% to 85%, in the B cell population from 26% to 67%, in the T cell population from 65% to 76%, and in the NK cell population from 28% to 40%, respectively (Figure 3, Table 1). However, no augmentation of expression intensity was observed in macrophage, B cells and NK cell populations (see comparable MFI in most cell populations, Table 1). Only T cells displayed a slight increase in the mean fluorescence intensity (Figure 3B, right and Table 1) whereas all other populations revealed a decrease. We also compared the cellular infiltration by flow cytometry of wild type and *Ifi27l2a* knock-out mice on day 3 p.i. and in non-infected controls. We did not find any significant difference in resting or activated macrophages, granulocytes, NK cells, T cells or B cells (data not shown).

**Table 1:** Analysis of *Ifi27l2a*-LacZ reporter gene expression by flow cytometry.

	Leuko (%)	FluoRep+ (%)	MFI	n
cell type	Macrophages (CD11b^high^)			
non-infected	13	59	11800	3
infected	32	85	8112	4
cell type	B cells (CD19)			
non-infected	33	26	6891	3
infected	24	67	6643	4
cell type	T cells (CD3)			
non-infected	26	65	8213	3
infected	15	76	10500	4
cell type	NK cells (NKp46)			
non-infected	15	28	17850	2
infected	20	40	6644	4

Lungs from infected and non-infected KO mice were harvested and homogenized as described in material and methods. After staining with specific antibodies for macrophages, B cells, T cells and NK cells the respective cell populations were analyzed for expression of the LacZ reporter gene using a fluorescent substrate (fluorescein di β-D-galactopyranoside, see material and methods for details). Leuko %: percentage of respective cell population (CD11b^high^, CD19, CD3 and NKp46) with respect to total leukocyte counts. Please note that the total percentages do not add up to 100% of the leucocytes since there are also immune cells that do not express any of the respective markers. FLuoRep+ %: percent of cells positive for the fluorescent substrate within the respective immune cell population. n: number of biological replicates per group. Median values are calculated for each group. MFI is the geometric mean of the Mean Fluorescence Intensity (here gated on the LacZ+ subpopulation of the respective cell type).

### *Ifi27l2a* knock-out mice do not exhibit a more severe phenotype or increased viral load after influenza A infection

**Figure 5:**
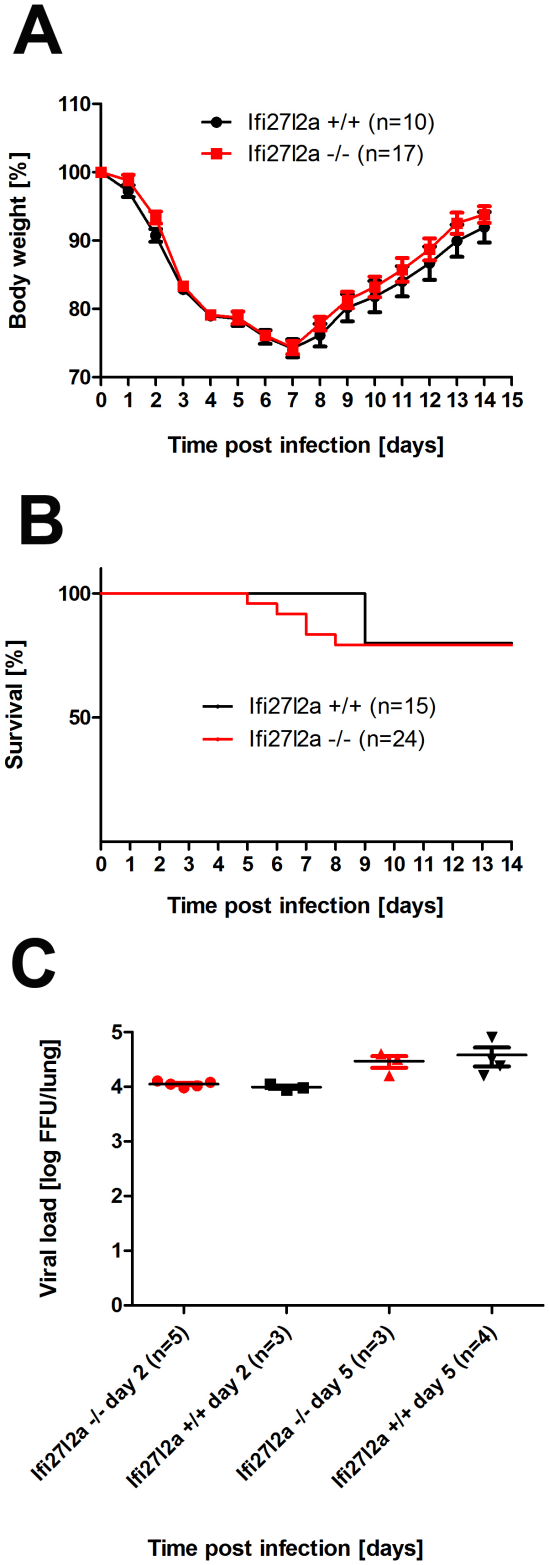
Comparison of body weight, survival and viral load between knock-out and wild type mice after infection with influenza A H1N1 virus. Female mice were infected with 2×10^5^ FFU PR8 (H1N1) virus and body weight loss (A) and survival (B) were monitored until day 14 p.i. Mice with a weight loss of more than 30% of the starting body weight were euthanized and recorded as dead. Viral load was determined in infected lungs at days 2 and 5 p.i. (C). Weight loss data represent mean values +/− SEM. Note that for the later time points, only data of surviving mice are presented. Body weight loss was not significantly different between wild type (C57BL/6N- *Ifi27l2a^+/+^*) and homozygous *Ifi27l2a^-/-^* mutant mice (using the Mann Whitney U test). Survival (using the log-rank test) and viral load in infected mice at days 2 and 5 after infection (using the Mann Whitney U test) were not significantly different between wild type and knock-out mice.

Next, we infected *Ifi27l2a^-/-^* knock-out mice with influenza A virus (PR8M, H1N1) to investigate if deletion of this gene resulted in increased susceptibility. Surprisingly, body weight loss as well as survival rate were not different from wild type mice (Figure 5A, B). Both genders, female and male mice were tested and no differences to control mice were observed (data not shown). In addition, viral loads in the lungs were not significantly different in wild type versus knock-out mice (Figure 5C). Furthermore, we ascertained body weight and survival after infection with H7N7 influenza A virus subtype (A/Seal/Massachusetts/1/1980 H7N7, SC35M). No significant differences were observed (Figure S4 in File S1) of *Ifi27l2a^-/-^* mice and controls. Immunohistochemical staining for influenza A virus in lungs from PR8 infected mice showed replication of virus in the bronchial epithelium at day 2 p.i. and also in alveolar regions at day 5 p.i. to the same extend in *Ifi27l2a^-/-^* and wild type mice (Figure S5 in File S1). H&E staining of lung sections revealed tissue destruction of epithelial cell layers and first infiltrating immune cells, mostly granulocytes, in peribronchial, perivascular and alveolar regions at day 2 p.i. in wild type mice (Figure S6A in File S1). The same phenotype was observed for *Ifi27l2a^-/-^* mutant mice (Figure S6D in File S1). At day 5 p.i., destruction of the epithelial layer and sloughing of cells into the lumen in many bronchioli as well as massive infiltrates of immune cells, including lymphoid cells, were observed in all regions of the lung. However, no obvious differences were detectable between wild type and *Ifi27l2a^-/-^* mice (Figure S6B, E, respectively in File S1). At day 8 p.i. only some sloughing of dead cells into bronchiolar lumen was seen in wild type mice but was still frequently found in *Ifi27l2a^-/-^* mutant mice. Infiltration of lymphoid immune cells was massive at day 8 p.i. in both wild type and knock-out mice (Figure S6C, F, respectively in File S1).

## Discussion

We studied expression of *Ifi27l2a* by RT-PCR and detected transcripts in kidney, liver, lung, spleen, and salivary gland. These results corroborate and extend previous microarray studies demonstrating expression of *Ifi27l2a* in salivary gland, adrenal gland, adipose tissues, spleen, lymph nodes, B cells and macrophages (GEO database record GSE10246). For a more detailed analysis of gene expression patterns at the cellular level, we used an *Ifi27l2a*-targeted allele in which the LacZ reporter was inserted into the *Ifi27l2a* locus. This allele allowed us to follow *Ifi27l2a-*positive cells directly in tissue sections and by flow cytometry. We concentrated on the lung tissue, since we were mainly interested in regulation of the *Ifi27l2a* gene before and after influenza A infection.

In non-infected mice, reporter gene activity could be detected in macrophages in the alveolar regions of the lung by histochemical analysis. Flow cytometric studies revealed expression of the *Ifi27l2a*-driven LacZ reporter gene in macrophages and also in T, B and to a marginal extend NK cells. After infection with influenza A virus, *Ifi27l2a* expression strongly increased in the whole lung [38], [43]. At the same time, the number of recruited immune cells increased in the infected lungs. However, no up-regulation of reporter gene expression was seen within individual cells of any type. We thus conclude that the total increase observed for *Ifi27l2a* transcripts in the whole lung after infection is mainly due to infiltration of *Ifi27l2a* expressing macrophages and lymphocytes but not to up-regulation of *Ifi27l2a* within individual cells.

Furthermore, no LacZ expression was observed in alveolar or bronchiolar epithelial cells. Immunofluorescent studies confirmed expression of LacZ reporter in alveolar macrophages and no expression in neutrophils. Although we did not detect LacZ expression in alveolar and bronchiolar epithelial cells, we have to note a strong cross-reactivity of the anti-ß-galactosidase antibody with endogenous ß-galactosidase, especially in bronchiolar cells [44], and therefore we cannot exclude a low level of *Ifi27l2a* expression in these cells. In summary, these results suggest that *Ifi27l2a* is neither expressed in infected epithelial cells nor in neighboring epithelial cells or granulocytes near infected areas. Expression of *Ifi27l2a* was not detectable in lymphocytes in histological studies but with the more sensitive method of flow cytometry. Therefore, infiltrating macrophages plus lymphocytes are the main source of increased *Ifi27l2a* expression in the lung.

In our *in vivo* studies, *Ifi27l2a* was neither induced to detectable levels in the infected bronchiolar epithelial cells nor in alveolar cells. These observations are different from *in vitro* studies showing that *Ifi27l2a* was strongly induced after treatment of cell lines and primary cell cultures with interferon, including vascular smooth muscle cells and a monocyte cell line [40]. Similarly, Wang et al. [45] and Ionnidis et al. [46] described the expression of genes, including *Ifi27l2a*, after influenza infection in differentiated AECII cells *in vitro*. However, up-regulation of *Ifi27l2a* in cultured cell lines after infection or interferon treatment may not necessarily mean that the gene is induced directly by interferons. Many chemokines and cytokines are also up-regulated, and therefore, the activation of *ifi27l2a* gene expression may well be explained by a secondary stimulation via other chemokines and cytokines. Innoidis et al. [46] also reported that peripheral blood monocytes from patients with acute influenza A or RSV respiratory bronchitis express *Ifi27l2a*. These observations are well in agreement with our results.

Furthermore, *Mx1* (myxovirus (influenza virus) resistance 1), an ISG which is directly activated by type I and III interferons [47], is mainly expressed in bronchiolar epithelial cells after influenza A infection. No *Mx1* expression was observed in macrophages [48]. These observations suggest that the main interferon production and activation of ISGs that are directly regulated by interferons after influenza A infection occurs in the bronchiolar epithelium. Infection of epithelial cells results in the secretion of long-range signaling molecules which in turn triggers the recruitment of *Ifi27l2a-*positive leukocytes to the infected lung. The increase in cell numbers of these cell populations constitutively expressing *Ifi27l2a* leads to the observed increase in the *Ifi27l2a* transcript levels in the lung. This scenario may be different in other tissues or after infections with different pathogens.

It should also be noted that Ifi27l2a-specific antibodies are not available and that only expression of the LacZ reporter gene can be followed. Thus, we cannot exclude that the LacZ reporter gene may not fully reproduce the expression of the endogenous gene locus. Also, we did not measure protein levels and we can thus not exclude regulation at the post-transcriptional or post-translational level. We also note that we cannot exclude low levels of expression that may be missed by our methods. For a final formal proof, more experiments using Ifi27l2a antibody staining or *in situ* hybridization studies would be necessary.

Previous reports describe the analysis of *Ifi27l2a* knock-out mice with respect to cardio-vascular disease [40] or bacterial sepsis [42]. However, *Ifi27l2a* deficient mice have not yet been studied in the context of viral infections. The original *Ifi27l2a^-/-^* mice were generated and maintained on a mixed background (50% 129S/v and 50% Swiss Webster, [40]). Since the 129 background itself renders mice highly susceptible to influenza A infections [49], we generated a new mutant line on a C57BL/6N background using a KOMP targeted ES cell line. Most surprisingly, *Ifi27l2a^-/-^* mutant mice did not exhibit an increased susceptibility to influenza A infections as one would expect based on previous transcriptome and network analyses [37]. The most likely explanation is that a large number of IFN-induced genes are activated after influenza A infection and that there is functional redundancy between them. Similar observations have been obtained in knock-out mice in which one of interferon receptors have been mutated. Deletion of the interferon type I receptor *Ifnar1* alone leads only to a moderate increase in susceptibility whereas deletion of both *Ifnar1* (interferon (alpha and beta) receptor 1) and the *Ifnlr1* (*Il28ra*, interferon lambda receptor 1) gene strongly increased susceptibility to influenza infections [48], [50].

Deletion of the *Ifi27l2a* gene renders knock-out mice more susceptible to septic shock after bacterial infection [42]. This is noteworthy in the context of our results where we did not observe an increased susceptibility to influenza A virus infections. These findings may be explained by the fact that during bacterial infections macrophages play important roles in the host defense, especially for sepsis [51], [52], [53].

In conclusion, our *in vivo* studies shed new light on the regulation and function of *Ifi27l2a* during influenza infections of the lung which were unexpected based on previous *in vitro* studies. We demonstrated that *Ifi27l2a* is expressed in immune cells of the lung before and after infection and that the increase in total expression levels after infection is mainly due to the constitutively expression of infiltrating immune cells. Furthermore, analysis of *Ifi27l2a* knock-out mice revealed no increased susceptibility to influenza virus suggesting a network of ISGs with redundant biological functions of the host response to influenza infections.

## Material and Methods

### Ethics statement

All experiments in mice were approved by an external committee according to the national guidelines of the animal welfare law in Germany (BGBl. I S. 1206, 1313 and BGBl. I S. 1934). The protocol used in these experiments has been reviewed by an ethics committee and approved by the ‘Niedersächsisches Landesamt für Verbraucherschutz und Lebensmittelsicherheit, Oldenburg, Germany’ (Permit Number: 33.9.42502-04-051/09).

### Virus, mice and plasmids

Original stocks of viruses were obtained from Stefan Ludwig, University of Münster (PR8, A/PuertoRico/8/34 H1N1, Münster variant, as described in [54]) and from Peter Stäheli, University of Freiburg (A/Seal/Massachusetts/1/1980 H7N7, SC35M [55]). Virus stocks were prepared by infection of 10-day-old embryonated chicken eggs as described in [56]. The *Ifi27l2a* knock-out mouse strain (C57BL/6NTac-*Ifi27l2a^tm1(KOMP)Vlcg^*) used for this research project was created from ES cell clone 10949A-H8, obtained from the KOMP Repository (www.komp.org)). Methods used for generation of KOMP mice have been published previously [57]. Animals were maintained under specific pathogen free conditions at the animal facility of the HZI in Braunschweig. Homozygous mutant mice were genotyped by PCR analysis and then used for infections. For genotyping, a set of three primers was used which generate a 348 bp fragment for homozygous mutant and heterozygous mice and a 557 bp band for heterozygous and wild type mice, respectively. Primer sequences used as forward primer: GACAGAGTTCAGGAAAGATG, and as reverse primers LacZRev GTCTGTCCTAGCTTCCTCACTG and Ifi27l2a inrev ACATTCATCTACTTGCTGCTC.

### RNA isolation, purification and RT-PCR analysis

Total RNA was prepared from lungs using the RNeasy Midi Kit (Qiagen, Hilden, Germany) following the manufacturer's protocol. Samples containing 500 ng of total RNA were then digested with DNase I (to remove any DNA in the preparation) and cDNA was synthetized using the BioscriptTM (Bioline GmbH, Germany). Subsequently, Taq polymerase (Genecraft Germany) and gene-specific primers (Table S1 in File S1) were used for PCR amplification. Amplified fragments were analyzed on a 1% agarose gel.

### Infection of mice and measurement of body weight loss and survival

For infection experiments, female and male mice at the age of 12–14 weeks were anesthetized by intra-peritoneal injection of Ketamin-Xylazine (5% each) solution in sterile NaCl (100 mg/ml Ketamine, WDT, Garbsen, Germany; 20 mg/ml Xylazine, Bayer Health-Care, Leverkusen) with a dose adjusted to the individual body weight (200 µl/20 g body weight). Infection was performed by intranasal application of virus solution in 20 µl of sterile phosphate-buffered saline. Subsequently survival and body weight loss were monitored until day 14 p.i. In addition to mice that were found dead, mice with a weight loss of more than 30% of the starting body weight were euthanized and recorded as dead.

### Determining of infectious viral particles

Viral load in infected lungs was determined on MDCK (Madin-Darby Canine Kidney) cells using the FFU assay as described [54]. Detection limit of the assay is at 40 infectious particles/lung. Thus, for samples where no virus was detected, the data points were set to 40 FFU/lung. For determination of viral load, lungs of mice were homogenized in phosphate buffered saline (PBS) with 0.1% BSA using the Poly Tron 2100 homogenizer. Debris was removed by centrifugation. The samples were stored in aliquots at −70°C. Serial 10-fold dilutions of lung homogenates in DMEM containing 0.1% BSA were prepared and viral titers determined by the FFU assay.

### Analysis of LacZ reporter gene expression in tissue sections

Whole organs of *Ifi27l2a^-/-^* and wild type mice were fixed with 0.5% glutaraldehyde in PBS for 15 minutes at room temperature, washed twice with PBS, and then stained overnight at 37°C in staining solution containing 1 mg/ml X-gal (Sigma Aldrich, St. Louis, MO, USA), 1 mM MgCl_2_, and 5 mM potassium ferrocyanide (Sigma Aldrich). Cryo-sectioning (10 µm) and subsequent LacZ staining were performed as described previously [58]. In brief, sections were fixed in 0,5% (v/v) glutaraldehyde/PBS for 10 minutes at 4°C, washed in 1 mM MgCl_2_/PBS and stained over night at 37°C in staining solution containing 1 mg/ml X-gal (Sigma Aldrich, St. Louis, MO, USA), 1 mM MgCl_2_, and 5 mM potassium ferrocyanide (Sigma Aldrich). The slides were counterstained with Nuclear Fast Red (Sigma) for 1 minute at room temperature.

### Histological, immunohistochemical and immunofluorescence analyses

Lungs were prepared and immersion-fixed for 24 hours in 4% buffered formaldehyde solution (pH 7.4), dehydrated in a series of graded ethanol and embedded in paraffin. Sections (0.5 µm) were cut from five evenly distributed levels of the paraffin blocks and stained with haematoxylin and eosin. For immunohistochemical studies, sections were stained with a polyclonal primary antibody (against influenza A H1N1 virions; Virostat) overnight at 4°C and subsequently tissue sections were incubated for 30 min with the secondary antibody (rabbit anti-goat-biotin; KPL; Gaithersburg, Madison, USA) and counterstained with haematoxylin. For immunofluorescence diagnosis 12 µm cryo-sections were air-dried, fixed in acetone at −20°C and rehydrated in PBS. Slides were blocked with 2% BSA in PBS and stained with following antibodies. Primary antibodies used were: Biotin anti-CD11b (1∶200, clone M1/70, Siegfried Weiß, HZI), anti-beta Galactosidase (1∶200, Abcam). Fluorochrome-conjugated secondary antibodies used were: Streptavidin-Alexa Fluor 488 (1∶200, Invitrogen), Anti-Mouse Ly-6G (Gr1) eFluor 660 (1∶200, eBiosciences), Alexa Fluor 555 goat anti-chicken IgY H&L (1∶500, Abcam) and APC anti-mouse CD326 (Epcam) (1∶200, BioLegend). After staining slides were washed with PBS, dried and mounted with Neo-Mount (Merck, Darmstadt, Germany). Analyses were performed using a Zeiss LSM510 laser scanning microscope with a 40× oil immersion objective. Appropriate negative controls without the primary antibodies were performed and no immunofluorescence was detected but a strong cross-reaction of the anti-beta-galactosidase antibody with endogenous beta-galactosidase was observed in bronchial epithelial cells.

### Flow cytometry

Lungs from mice were harvested and homogenized by passing through a 100 µm cell strainer (Becton Dickinson) and resuspended in PBS followed by a density centrifugation with lympholyte M (Cedarlane Laboratories) at RT. The interphase was extracted and washed with PBS/3%FCS buffer. Cells were processed according to the manufacturer's protocol (Molecular Probes, FluoReporter lacZ Flow Cytometry Kit): Cells were resuspended in 100 µl “staining medium” (PBS, 4%FCS, 10 mM HEPES, pH 7.2) and incubated at 37°C for 10 minutes. In parallel “working solution” including fluorescein di β-D-galactopyranoside (FDG), 2 mM in ddH2O, has to be incubated at 37°C for 10 minutes. An equal amount of “working solution” has to be added to the cell suspension and was incubated for 2 minutes at 37°C. FDG loading was stopped by 1∶10 dilution with ice cold “staining medium” including 1.5 µm propidium iodide. Cells were kept on ice during the subsequent staining procedure: Monoclonal antibodies (α-NKp46 PerCp eFluor710, α-CD3 Alexa 647, α-CD19 APC, α-CD11b, α-CD45 FITC, α-F4/80 PerCP) were added to the respective cell suspensions after blocking unspecific binding with 2.4G2 (α-CD16/32), incubated 30 minutes at 4°C washed twice with “staining medium” and measured on an Accuri C6 (Becton Dickinson) or a FACScalibur (Becton Dickinson). Cells from non-infected wild-type mice did not reveal any significant staining (data not shown). Further detailed analyses were performed with FlowJo 7.6.4.

### Statistical analysis

For statistical analysis, GraphPad Prism 5.0 software was used. Results were presented as means ± SEM. Statistical significance between groups were determined using the Mann-Whitney U test for body weight loss curves. The log-rank test was used to determine significant differences of the survival curves.

## Supporting Information

File S1 **Supporting information.** Figure S1: Ifi27l2a is expressed in several tissues in wild type mice. Figure S2: Ifi27l2a is up-regulated in lung tissue after infection. Figure S3: Schematic illustration of targeted Ifi27l2a gene locus. Figure S4: Comparison of body weight and survival between knock-out and wild type mice after infection with influenza A H7N7 virus. Figure S5: Virus spread in infected lungs of Ifi27l2a knock-out and wild type mice. Figure S6: Lung pathology in infected Ifi27l2a knock-out and wild type mice. Table S1: Primers for RT- PCR analysis.
(PDF)
