# Supplementary material for: The Interferon-Induced Gene Ifi27l2a is Active in Lung Macrophages and Lymphocytes After Influenza A Infection but Deletion of Ifi27l2a in Mice Does Not Increase Susceptibility to Infection
Source: PLoS One. 2014 Sep 3;9(9):e106392. doi: 10.1371/journal.pone.0106392 (PMC4153650; doi:10.1371/journal.pone.0106392)
Supplement: File S1 — Supporting information. Figure S1: Ifi27l2a is expressed in several tissues in wild type mice. Figure S2: Ifi27l2a is up-regulated in lung tissue after infection. Figure S3: Schematic illustration of targeted Ifi27l2a gene locus. Figure S4: Comparison of body weight and survival between knock-out and wild type mice after infection with influenza A H7N7 virus. Figure S5: Virus spread in infected lungs of Ifi27l2a knock-out and wild type mice. Figure S6: Lung pathology in infected Ifi27l2a knock-out and wild type mice. Table S1: Primers for RT- PCR analysis. (PDF) [file pone.0106392.s001.pdf]

Supplement data

The interferon-induced gene *Ifi27l2a* is active in lung macrophages and lymphocytes after influenza A infection but deletion of *Ifi27l2a* in mice does not increase susceptibility to infection

Mohamed A. Tantawy[a,b,c], Bastian Hatesuer[a,c], Esther Wilk[a,c], Leonie Dengler[a], Nadine Kasnitz[d], Siegfried Weiß[d], and Klaus Schughart[a,e,f]

Author affiliations:

[a]Department of Infection Genetics, Helmholtz Centre for Infection Research and University of Veterinary Medicine Hannover, [d]Department of Molecular Immunology, Helmholtz Centre for Infection Research, and [e]University of Tennessee Health Science Center

[c]Equal contribution as first author

[b]Present address: Institute for Experimental Infection Research, TWINCORE Center for Experimental and Clinical Infection Research, Hannover

[f]Author for correspondence at: Klaus Schughart, Department of Infection Genetics, Helmholtz Centre for Infection Research, 38124 Braunschweig, Germany; e-mail: klaus.schughart@helmholtz-hzi.de; Tel: +49-531-6181-1100; FAX: +49-531-6181-1199

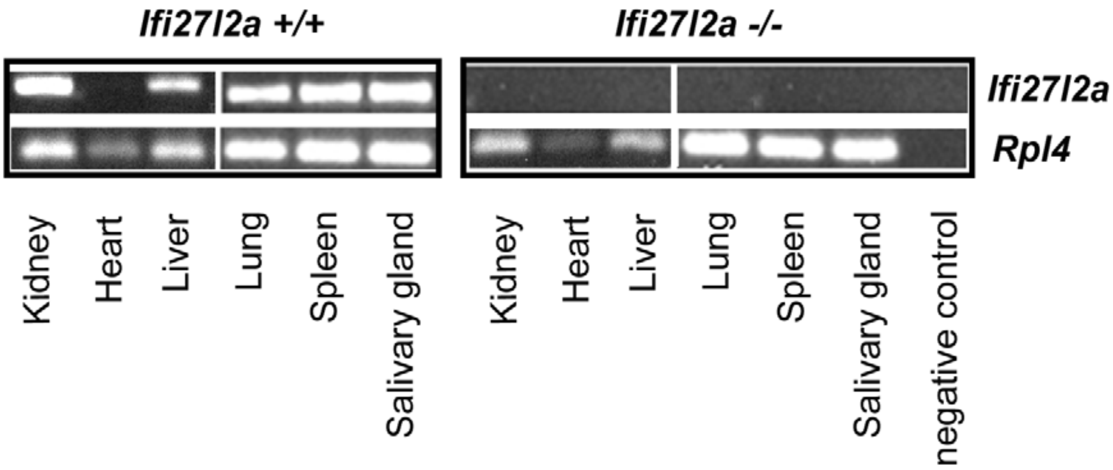

Figure S1. *Ifi27l2a* is expressed in several tissues in wild type mice and absent in *Ifi27l2a* knock-out mice.

RNA was extracted from different tissues of wild type C57BL/6N-*Ifi27l2a*$^{+/+}$ or *Ifi27l2a*$^{-/-}$ mice and amplified by RT-PCR. Amplification of the housekeeping gene *Rpl4* was used for RNA normalization. As negative control no RNA was used in the RT reaction. In *Ifi27l2a* knock-out animals no signals could be detected in any organs that were analyzed. Also, no expression of *Ifi27l2a* was seen in *Ifi27l2a*$^{-/-}$ mice after influenza infection (data not shown)

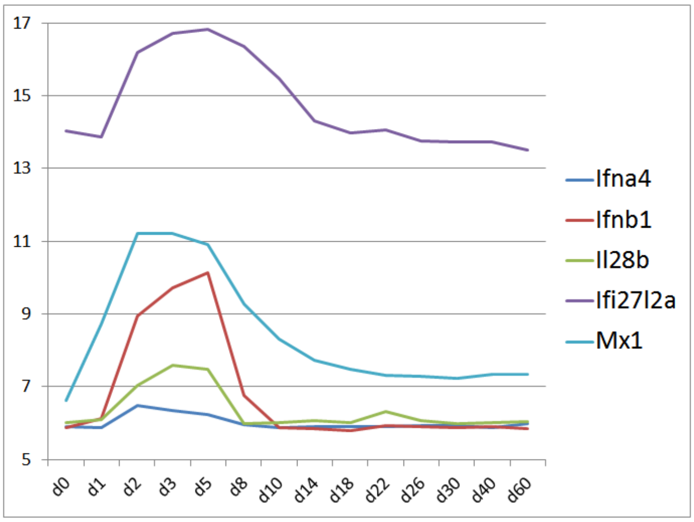
17
15
13
11
9
7
5
d0
d1
d2
d3
d5
d8
d10
d14
d18
d22
d26
d30
d40
d60
Ifna4
Ifnb1
Il28b
Ifi27l2a
Mx1

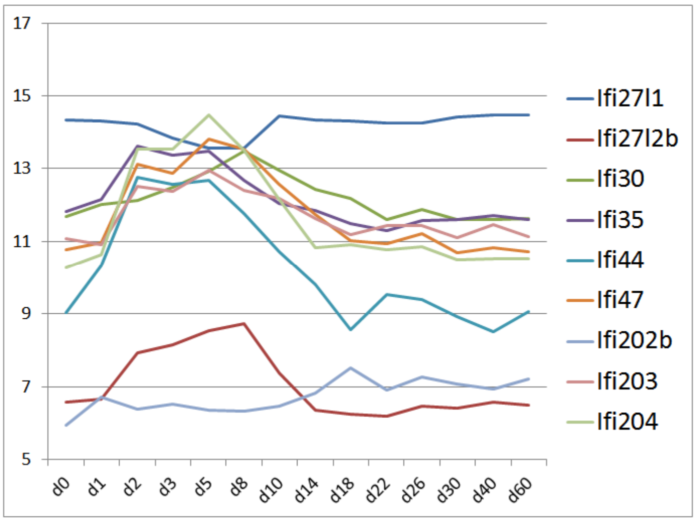
17
15
13
11
9
7
5
d0
d1
d2
d3
d5
d8
d10
d14
d18
d22
d26
d30
d40
d60
Ifi27l1
Ifi27l2b
Ifi30
Ifi35
Ifi44
Ifi47
Ifi202b
Ifi203
Ifi204

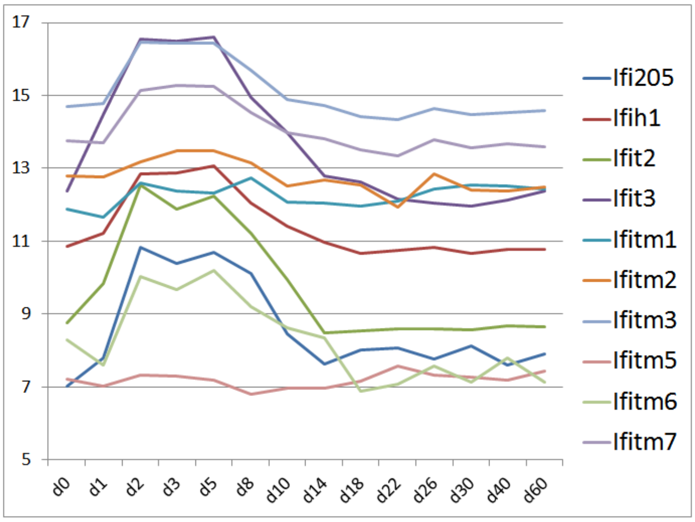
17
15
13
11
9
7
5
d0
d1
d2
d3
d5
d8
d10
d14
d18
d22
d26
d30
d40
d60
Ifi205
Ifih1
Ifit2
Ifit3
Ifitm1
Ifitm2
Ifitm3
Ifitm5
Ifitm6
Ifitm7

32

Figure S2. *Ifi27l2a* is up-regulated in lung tissue after infection.

Expression levels of *Ifnb1*, *Ifna4* and different interferon-induced genes in the lung after infection of female C57BL/6J mice with $2x10^3$ FFU PR8 (H1N1) virus are represented, using data from a previously published microarray study [1]. *Ifi27l2a* (upper panel) is already expressed in non-infected lungs at a high level and strongly up-regulated after influenza infection from day 1 until day 8, together with up-regulation of *Ifnb1* and *Ifna4. Ifi27l2a* displays the same expression kinetics as the interferon-induced *Mx1* gene. In cases, where multiple probesets per gene were analyzed, we selected the one which exhibited the highest total expression signal. x-axis: days post infection, y-axis: expression levels (microarray intensity signal) on a log_2 scale; d0: mock-infected mice.

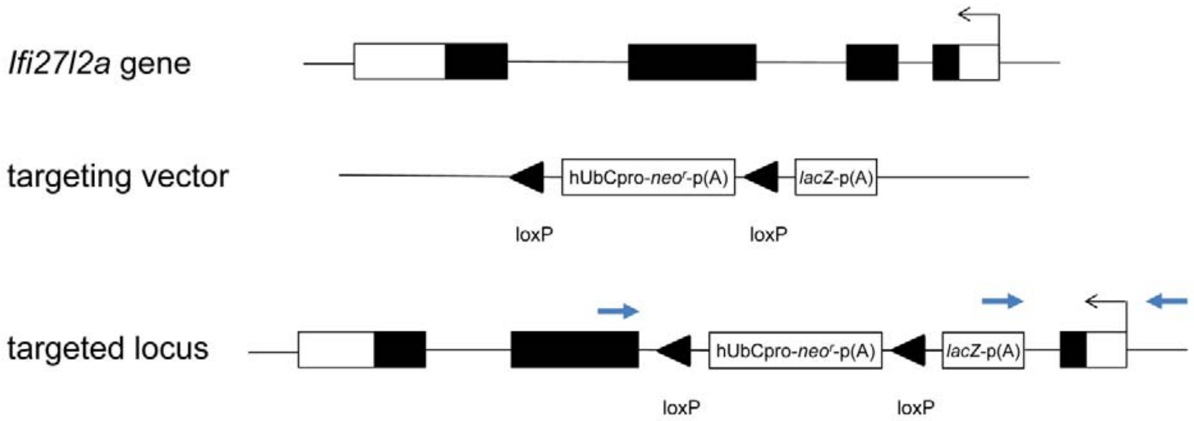

Figure S3. Schematic illustration of targeted *Ifi27l2a* gene locus. hUbCpro: promoter of the human ubiquitin C gene, *lacZ:* ß-galactosidase coding sequence from the *E.coli lacZ* gene, *Neo^r:* coding sequence for neomycin phosphotransferase, p(A): polyadenylation signal, arrows: direction of gene transcription, blue arrows: PCR primer, black boxes: *Ifi27l2a* coding region.

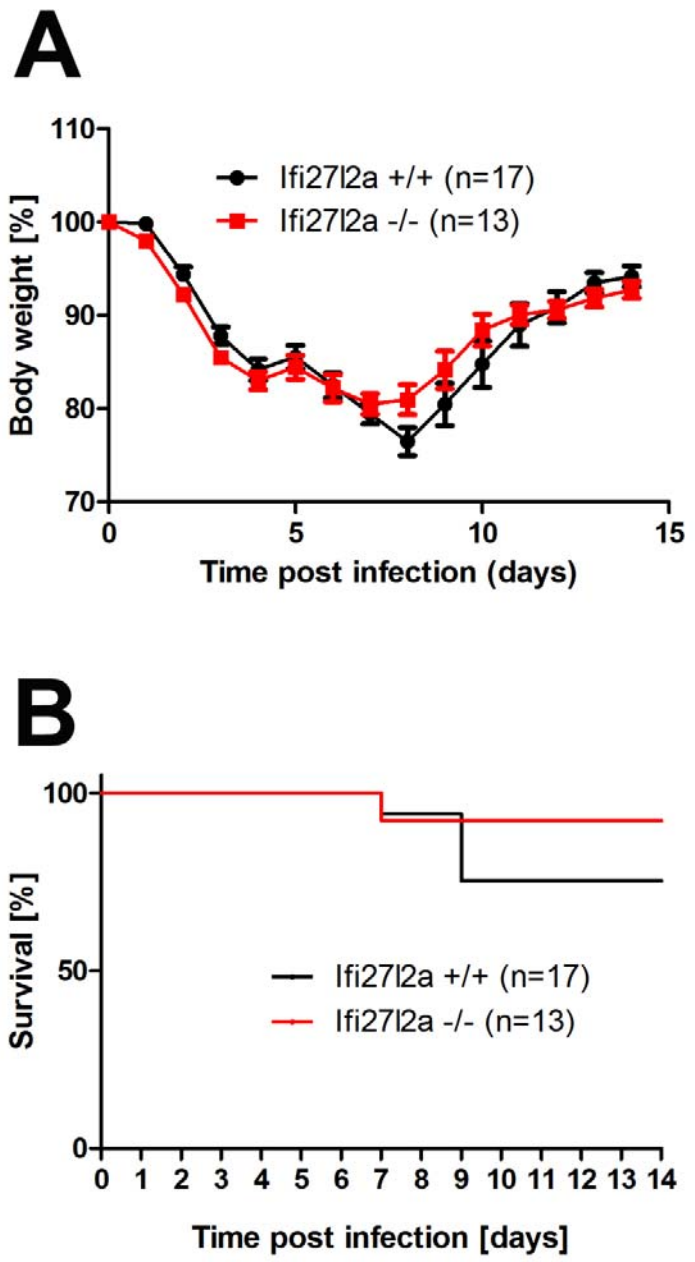

Figure S4. Comparison of body weight and survival between knock-out and wild type mice after infection with influenza A H7N7 virus.

Female mice were infected with $2x10^4$ FFU SC35M (H7N7) virus and body weight loss (A) and survival (B) was monitored until day 14 p.i. Mice with a weight loss of more than 30% of the starting body weight were euthanized and recorded as dead. Weight loss data represent mean values +/- SEM. Note that for the later time points, only data of surviving mice are presented. No

significant differences could be observed with respect to body weight loss and survival between wild type (C57BL/6N-*Ifi27l2a*$^{+/+}$) and *Ifi27l2a*$^{-/-}$ homozygous mutant mice using the Mann Whitney U test for body weight loss and the log-rank test for survival.

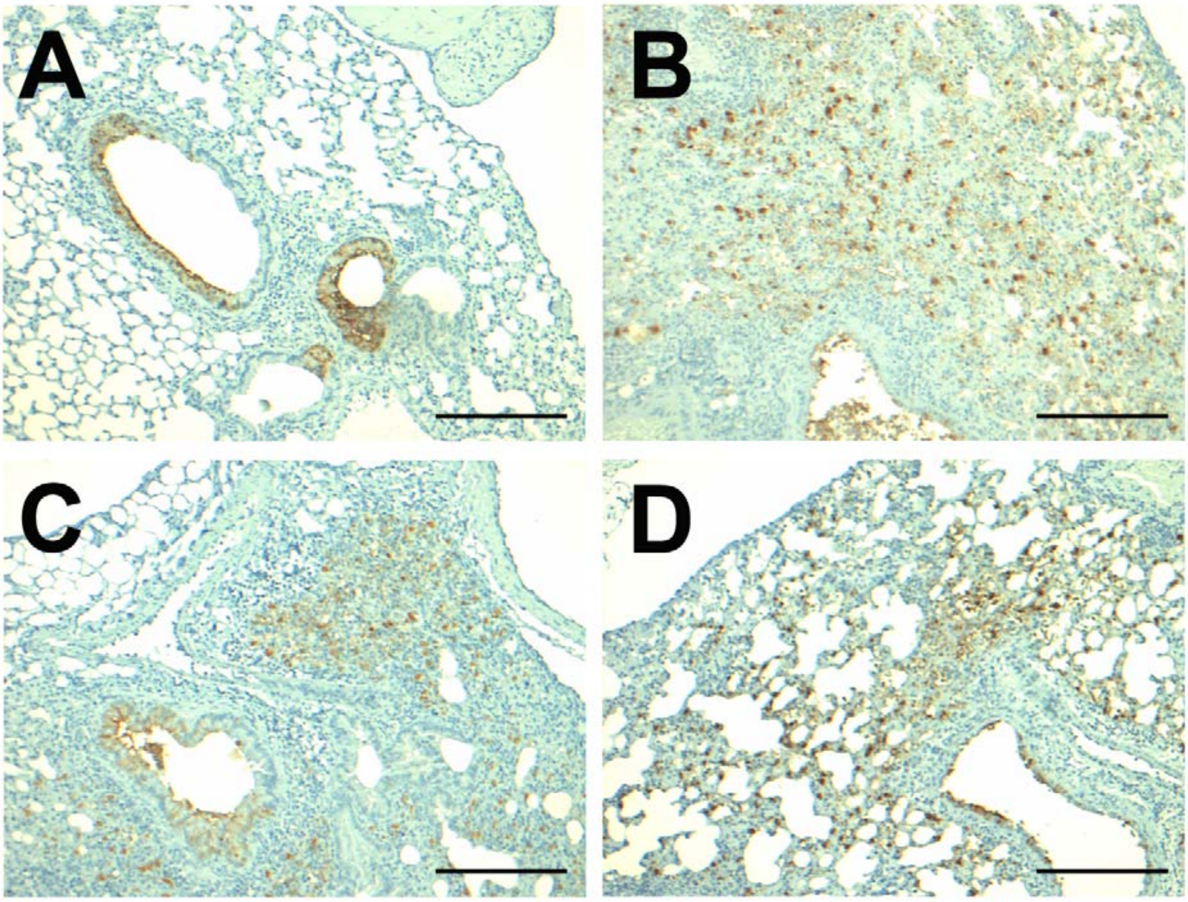

Figure S5. Virus spread in infected lungs of *Ifi27l2a* knock-out and wild type mice.

Wild type (C57BL/6N-*Ifi27l2a*$^{+/+}$; A, B) and *Ifi27l2a*$^{-/-}$ (C, D) female mice (n=3) were infected with $2×10^5$ FFU PR8 virus. Lungs were prepared at day 2 p.i. (A, C) or day 5 p.i. (B, D), embedded in paraffin and lung sections were then processed with antibodies against influenza A H1N1 virions to detect infected cells. Antigen-positive cells are stained brown. No obvious difference was seen between knock-out and wild type mice. Sections were counterstained with hematoxylin. Bars: 200 µm.

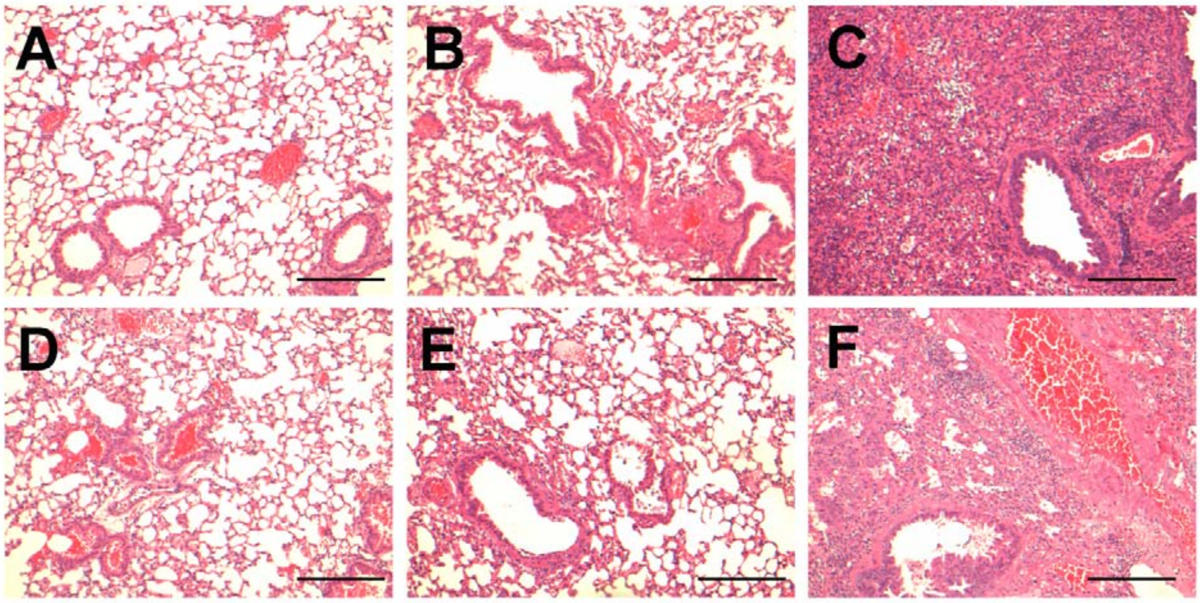

Figure S6. Lung pathology and immune cell infiltrations in infected *Ifi27l2a* knock-out and wild type mice

Wild type (C57BL/6N-*Ifi27l2a*$^{+/+}$; A, B, C) or *Ifi27l2a*$^{-/-}$ (D, E, F) female mice (n=3) were infected with 2×10^{5} FFU PR8 virus and lung sections from day 2 p.i. (A, D), day 5 (B, E) or day 8 p.i. (C, F) were stained with hematoxylin and eosin (H&E). No obvious differences were observed between *Ifi27l2a* knock-out and wild type mice. Bars: 200 µm.

Table S1: Primers for RT- PCR analysis

Gene name	Forward primer	Reverse primer
Ifi27l2a RT	CTGTTTGGCTCTGCCATAGGAG	CCTAGGATGGCATTTGTTGATGTGG

References

1. Pommerenke C, Wilk E, Srivastava B, Schulze A, Novoselova N, et al. (2012) Global transcriptome analysis in influenza-infected mouse lungs reveals the kinetics of innate and adaptive host immune responses. PLoS ONE 7: e41169.
